# Medical and Legal Aspects of Child Sexual Abuse: A Population-Based Study in a Hungarian County

**DOI:** 10.3390/ijerph15040701

**Published:** 2018-04-09

**Authors:** Andrea Enyedy, Panagiotis Tsikouras, Roland Csorba

**Affiliations:** 1 Department of Obstetrics and Gynecology, City Hospital of Nyíregyháza, Mák u. 10–14, 4400 Nyíregyháza, Hungary; enyedy.andrea@szszbmk.hu; 2 Department of Obstetrics and Gynecology, Faculty of Medicine, Democritus University of Thrace, 68100 Alexandroupolis, Greece; 3 Department of Obstetrics and Gynecology, City Hospital of Aschaffenburg, Am Hasenkopf 1, 63739 Aschaffenburg, Germany; drcsorbaroland@gmail.com; 4 Department of Obstetrics and Gynecology, Faculty of Medicine, University of Debrecen, 4033 Debrecen, Hungary

**Keywords:** child sexual abuse, adolescents, girls, boys, gender differences, legal proceedings, child-friendly justice, prevention

## Abstract

Background: Very few studies focus on childhood sexual abuse in middle European countries. Aim: The purpose of our study is to describe the medical and legal characteristics of children who experience sexual abuse and explore common features that may result in strategies for prevention. Methods: Between 2000 and 2015, 400 girls and 26 boys under the age of 18, suspected of being sexually abused, visited one of the four hospitals in a Hungarian county. Results: Mean age at onset was 10.81 years for boys, 13.46 years for girls. In 278 cases (65.3%), the perpetrator was known to the victim, and a stranger was suspected in 148 cases (34.7%). In 79 cases (30.7% of boys and 17.7% of girls), a family member was the accused perpetrator. In more than one-third (boys) and in one-fifth (girls) of cases, sexual abuse had occurred on multiple occasions. In the case of boys, child and adolescent sexual abuse (CSA) included oral genital, genital touching and genital to genital contact in 14 cases (53.8%) and anal intercourse in 12 (46.2%) cases. In case of girls, sexual abuse included coitus in 219 (54.8%), oral genital, genital touching, genital to genital contact in 164 (41.0%), anal abuse in 14 (3.5%) cases, physical injury was incurred in 15 cases. Legal proceedings followed the CSA in 205 (48.1%) cases. Conclusion: The results highlight the urgent need to address the issue of sexual abuse in Hungary and minimize its impact. Prevention requires a systematic and lifelong approach to educating children about personal space safety and privacy to reduce vulnerability and is the responsibility of parents and professionals.

## 1. Introduction

Child and adolescent sexual abuse (CSA) is a widespread problem not only in the western countries but also in the eastern and middle European countries, as well. Sexual abuse in childhood and adolescence is defined as the involvement of the young individual in a sexual activity that he/she does not fully comprehend or is not developmentally prepared for, or violates the law or social taboos of the society [1,2]. The sexual activities may include all forms of oral-genital, genital, or anal contact by or to the child, or nontouching abuses, such as exhibitionism, voyeurism, or using the child in the production of pornography. Sexual abuse includes a spectrum of activities ranging from rape to physically less intrusive sexual abuse. ‘Child sexual abuse is more frequent than childhood cancer, juvenile diabetes, and congenital heart disease combined...’ [3].

Various studies have researched the prevalence of child sexual abuse and the international estimates show prevalence rates that are frightening high. It is estimated that 10–20% of girls and 5–10% of boys are victims of child sexual assault [4], according to data collected between 1994 and 2007 in 39 predominance studies from 28 countries. These figures are in accordance with those of previous surveys [5]. In a meta-analysis of 323 studies worldwide, featuring a total of 9.9 million abused children, the worldwide incidence was revealed to be 12.7% (18.0% for girls, 7.6% for boys) [6]. In the USA, where the reporting of child sexual assault is obligatory, 60,000 to 80,000 confirmed cases are reported per year, showing a general decrease [7]. The incidence of child sexual assault is 20% in the UK and Switzerland [8,9], while it is to a certain degree higher in Germany, with 20.1%, and in Spain, with 22% [10,11]. In north European countries, the incidence appeared to be significantly lower: 17% in Norway and 13% in Sweden [12,13].

The literature reports a lifelong association between sexual victimization in childhood and adolescence and chronic mental and physical illness in adulthood [14]. CSA has been related to several negative health problems, including both physical and mental conditions [15,16,17]. Victims with child maltreatment histories are more likely to manifest greater risk for violent crimes [18], substance use disorders [19], more school discipline problems and suspensions [20], poor long-term intellectual and academic achievement [21], and greater likelihood of becoming a teenage parent [22,23]. There are also significant economic and societal consequences [24]. Women with a history of CSA account for a significantly higher proportion of primary care and outpatient costs than women without any such history [25].

There are limited studies that focus on childhood abuse in middle and eastern European countries. The available data from Hungary are inadequate and in many cases unreported; trustworthy data on the frequency of subtypes of sexual abuse do not exist.

There are no Hungarian National or Country statistics obtainable. The incidence and nature of child sexual abuse in Hungary have not been researched, apart from a recent study that investigated sexual abuse in only female children [26] and some unscientific evidence from split sources.

In Hungary, when a disclosure occurs, the emergency, gynecology and pediatric departments are commonly the portal for entry, and gynecologists (for female victims), pediatricians, and traumatologists (for male victims) are generally the ‘first reporters’ when sexual abuse is suspected. This is followed by reporting and the involvement of social services and law enforcement. Although reporting of child abuse is not mandatory, in the case of a victim under 18 years of age, legal proceedings can be initiated by the victim’s parent, carer or guardian, but not by the doctor.

The aim of this population-based study was to collect data from a Hungarian county with regard to the characteristic features of both male and female child abuse cases in an effort to further understand the extent of the problem, to progress towards reporting, and to approach the services and suggest educational and prevention strategies.

## 2. Methods

Our population-based study took place in 1 out of 19 Hungarian counties (Szabolcs-Szatmár-Bereg County, inhabitans: 585,000). In this county, there are only four cities, with four hospitals where these cases can be seen. The case notes of sexually abused girls and boys under 18 years, seen at the four hospitals of this county (University Teaching Hospital of Nyíregyháza, University Teaching Hospital of Fehérgyarmat, City Hospital of Mátészalka and the City Hospital of Kisvárda) between 1 January 2000 and 31 December 2015, were reviewed. These are the departments in Szabolcs-Szatmár-Bereg County to which adolescent cases with a suspicion of sexual abuse are referred. The female cases are exclusively referred to the gynecological department of the above listed hospitals; the boys are seen at the pediatric, urological and traumatological departments of these hospitals. Data were collected, recorded and extracted from the files of children who had allegedly experienced CSA and received care and follow-up services. Guidelines for standard management, including the definition of CSA and the purpose and procedure for the examination, were elaborated in detail at these departments.

In our population-based study, sexual abuse includes non-contact sexual abuse, sexual touch, oral sex, sexual penetration, anal sex and sexual exploitation.

The data collected included the age and education of the victim, family relationship between victim and perpetrator, frequency of abuse, type and place of abuse, season of the year and time of day when the abuse occurred, family relationship between victim and person accompanying her/him on presentation at the hospital, the time interval between alleged sexual contact and clinical examination and the findings on the medical examination.

An adolescent gynecologist for females, or a pediatrician, urologist or a traumatologist for males, performed the examination. All cases were managed according to the standard guidelines [27]. We obtained full medical and social histories and conducted physical examinations following a standard protocol. The clinical investigation included a ‘head-to-toe’ physical examination, assessment of sexual development, identification of any injury with specific reference to colposcopic appearances of the hymenal membrane and surrounding surfaces (girls), identification of any injury on the penis, scrotum and anus (boys), signs of abuse. Diagnosis of a possible pregnancy was ruled out by a urine test or ultrasound examination, collection of forensic evidence (sperm, saliva, other trace evidence) and evaluation for sexually transmitted infections were also part of our examination. Appropriate medical or surgical treatment was provided based on clinical findings. All relevant findings were documented and incorporated into a report and distributed in response to official requests. We analyzed the difference between CSA and rape, emphasizing the diagnostic challenges. The results of the legal proceedings were also recorded. The criminal, judicial and medical records were continuously monitored and compared.

Descriptive analyses were conducted using SPSS (Statistical Package for Social Science, SPSS Inc., Chicago, IL, USA). Estimates of mean value, standard deviation, frequency, *t*-test, Mann-Whitney test, confidence interval and correlation were assessed. Prevalence rates were tested using Pearson’s *Χ*^2^ tests. The significance level was kept at 0.05 level.

## 3. Results

Between 2000 and 2015, we enrolled 400 girls and 26 boys under 18 years of age who were seen at one of the four hospitals following sexual abuse in the study. Mean age at onset was 10.81 years (SD 3.453) for boys, 13.46 years (SD 3.364) for girls and 13.30 years (SD 3.425) for total victims. The sample characteristics are summarized in Table 1. Forty-four percent of children were aged between 11 and 14 years, the majority of boys (46.2%) were younger than 10 years old, the majority of girls (44.5%) were between 11 and 14 years old. Of the victims, 243 (57.0%) were pupils, 26 (6.1%) preschool children and 157 (36.9%) were working or unemployed. In 278 cases (65.3%), the perpetrator was known to the victim, and a stranger was suspected in 148 cases (34.7%). In 79 of all cases (30.7% by boys and 17.7% by girls) a family member was the accused perpetrator. Perpetrators were most often familiar to the children (38.5% for boys and 47.3% for girls). A relatively low proportion of the intrafamilial abuses were committed by the victim’s cousin or stepbrother (3–3 cases) for boys and the stepfather (24 cases) or father (12 cases) for girls. The distribution of the accompanying persons is presented in Table 1. In most cases, male victims were escorted to the hospital by their mother (9, 34.6%), and female victims by a police officer (200, 50.0%).

The characteristics of the CSA itself are presented in Table 2. Analysis of the time interval between the crime and the medical assessment showed a significant difference between the two genders. In case of male victims, only 2 victims (7.7%) had been able to get immediate emergency care, while 5 (19.2%) boys were seen at the hospital within 72 h and the majority—19 (73.1%)—were examined more than 72 h after the abuse. In case of female victims, the majority of the girls—152 (38.0%)—were seen more than 72 h after the abuse, but 98 (24.5%) cases had been able to receive immediate care, and 150 (37.5%) cases within 72 h. Furthermore, we found that in more than one-third (boys) and in one-fifth (girls) of cases, sexual abuse had occurred on multiple occasions. In the case of boys, CSA included sexual perversion (oral genital, genital touching and genital to genital contact) in 14 cases (53.8%) and anal intercourse in 12 (46.2%) cases; in our sample, the boys had not reported physical abuse. In the case of girls, sexual abuse included coitus in 219 (54.8%), sexual perversion in 164 (41.0%), and anal abuse in 14 (3.5%) cases; physical injury was incurred in 15 cases. Physical examination of the children focused on two points: sign of injury and identification/collection of forensic evidence whether genital, anal or extragenital. Injury occurred in 3.5% of cases, but none of them required surgical treatment. The presence of sperm was confirmed in 118 cases (27.7%). In 12 (3.0%) cases, the pregnancy test was positive. On looking at the diurnal timing of individual cases, we found dissonant results between the genders: the boys were more likely abused in the afternoon, while the girls were mostly abused in the evening. Seasonal occurrence showed comparable results, with CSA occurred mostly during the summer, 10 cases (38.5%) for boys, 117 cases (29.3%) for girls, when children were on holiday from school. In the majority of the cases (31%), the location of the abuse was the victim’s home (34.6% for boys and 30.85 for girls). Other locations included: at a familiar home (19.5%), public space (14.3%), in a forest/field (11%) and at a children’s home (4%). During the study period, legal proceedings followed the CSA in 205 (48.1%) cases. The number of perpetrators who were sentenced was 41 (9.6%), 22 of them were found guilty on charges of rape, 4 on charges of sexual perversion and 15 on charges of illegal sexual intercourse.

## 4. Discussion

Health care, child protection and legal systems continue to be challenged by the recognition of the societal scourge of child sexual abuse and strive to understand its complexities and how best to meet the myriad of interdisciplinary challenges. For more than 30 years, in many countries, medical doctors have been charged with the diagnosis, treatment and management of victims of sexual abuse. Their effort has been generally independent of the larger systems needed to provide protection, prevention and prosecution.

Hungary has just begun to deal with this societal issue. To the best of our knowledge, this is the first Hungarian population-based study to have researched characteristics of CSA for both girls and boys. We have been working to address the common myths regarding CSA and raise public awareness that CSA in Hungary can no longer to be ‘another hidden pediatric problem’, as stated by Kempe in 1977 [28].

In Hungary, during recent decades, child sexual abuse has become a priority for medical and criminal law professionals due to its frequent prevalence, serious adverse health effects and potential for lifetime consequence for the victim. In Hungary, when a disclosure occurs, the emergency, gynecology and pediatric departments are commonly the portal for entry, and gynecologists (for female victims), and pediatricians and traumatologists (for male victims), are generally the ‘first reporters’ when sexual abuse is suspected. Reporting and engagement of social services and law enforcement brings resources and critically important collaboration. Despite the robust association between gynecological symptoms and a history of CSA, no population-based central/eastern European data are available. Knowledge of the exact number of sexually abused children and the establishment of a systematic approach to address the medical and legal needs of alleged child victims are yet to be developed.

We were able to identify differences between CSA and rape. In the majority of cases (96.5%), force had not been used: children were mostly abused by perpetrators (65.3%) they knew and trusted. In one-third (boys) and one-fifth (girls) of the victims, the accused perpetrator of CSA was a family member and it was a domestic sexual attack (31%). In these cases, because of the long time interval between the sexual contact and the revelation (40.1%), the chance of identifying forensic evidence was lost. The time that passes between the abusive event and the physical examination is of great importance. Most sexually abused children will not have signs of genital or anal injury, especially when examined non-acutely.

Children who may have been abused should be examined by a physician to diagnose and treat any effect of the alleged sexual contact, which includes the identification of injuries, treatment for sexually transmitted infection (STI) and the collection of forensic evidence if present. The reality is that less than 5% of children will have diagnostic findings of sexual contact, and 3–5% will have an STI. Biological evidence (sperm) of recent abuse can be successfully secured (abuse within the past 24 h if before puberty, within the past 72 h in pubertal girls), and for medical reasons if there is any bleeding [29]. The most available evidence is what the child victim provides in their account of the inappropriate sexual contact.

As many as 30.8% of boys and 20% of girls reported histories of multiple occurrence of sexual contact. A delayed disclosure is typical for abuse, and since most injuries that children incur as a result of sexual contact will be superficial and heal without any lasting residual, most medical examinations show neither acute nor healed findings [30]. It has been proved that a single incomplete hymenal rupture can heal in 9 days, and a complete rupture in 24–30 days, after the trauma [31]. The most important reason for the paucity of abnormal findings is the nature of the abuse itself, as most perpetrators have little intent of actually harming the child, and if they do, the injuries are generally superficial. Although many nonmedical professionals may believe that it should be possible for a doctor to determine if a child has been ‘penetrated’, this is not always the case once children enter puberty and the hymenal membrane increases its elasticity and distensability. Determining ‘penetration’ in the prepubertal child is less of a challenge because of the lack of elasticity of the hymen, and when true vaginal penetration occurs, there is an accompanying history of pain, bleeding and diagnostic residual. That said, most prepubertal children experience penetration into the vaginal vestibule and not the vagina. Illustrative of the challenge of determining whether a pubertal child is a virgin is confirmed by a study in which only 2 (6%) of 36 pregnant teenagers manifested clear evidence of a prior penetration injury, and only 4 (11%) had suspicious, though not definitive, findings: “‘Normal’ does not mean ‘nothing happened’” [32]. Normal findings are the rule, not the exception, in victims of child sexual abuse, with or without penetration, whether chronic or acute [33,34,35].

Special attention is required in cases in which a member of the child’s family carried out sexual abuse. During the study-period, we saw only 8 male (30.7%) and 71 female (17.7%) cases in which the first instances of abuse were reported. The highest frequency was observed under the age of 10 years for boys and between the ages of 11 and 14 years for girls. The actual number was probably much higher than 79, because of underreporting. In almost every case, the child reported threats to maintain secrecy including threats of physical abuse if they disclosed. The mothers feared losing their homes, and possibly their children. This is the foundation on which multiple and chronic sexual abuse can continue. Most of the CSA cases occurred during the summer months when children were on holiday from school. Conversely, those children who were the victims of rape perpetrated by strangers (30.8% by boys and 35% by girls) presented immediately, and with more obvious findings (sperm found in 118 cases), as they had family support and immediate examination could be performed (in 100 cases, 23.5%). Unfortunately, few children reveal sexual contact immediately following CSA, which limits the opportunity to recognize injuries and collect forensic evidence. Sexual abuse is usually a chronic, complex, and often particularly traumatizing incident for the victim, frequently committed by family members or other trusted persons in the setting of relationship dependence and strong authority relationships [36].

In Hungary, since the decision made by the constitutional court on 4 September 2002, the perpetrator and the victim in CSA cases can be of the same sex. With that decision, the Law took account of the results of reported studies in male victims. In the case of a victim under 18 years of age, legal proceedings can be initiated by the victim’s parent, carer or guardian. The new act (from 1 July 2003) was intended to ensure considerate behavior towards the victim. The evidence given by the victim can be recorded on videotape and this tape can be used later during legal proceedings. In 2012 a new Penal Code was introduced dealing with sexual crimes. The essence of the legal change was that where the previous Penal Code had focused on the defense of gender morality and public interest, the new Penal Code focused on the individual, gender integrity and gender self-determination. None of the laws prescribe obligation of charges, but the ordinances of the private proposal and the desuetude of criminality have been changed. In 2012 our government announced the Year of Child-friendly Justice; within the framework of the National Court of Justice, a work-team was established in order to evolve the concept of child-friendly justice. The elements of child-friendly justice have been continuously becoming the part of legal practice. In Hungary, the greatest challenge is not only to create the Penal Code, but also to assure its enforcement. The experience of child sexual abuse has the potential to result in long-term emotional and behavioral consequences for the victim. When children are suspected of experiencing sexual victimization, they deserve professionals who are knowledgeable, skilled, sensitive professionals capable of formulating objective opinions that do not further betray their trust, allowing justice to be served.

In our study, only 205 (48%) of 426 cases had legal proceedings initiated. Delivery of the judgements against the 41 (9.6%) perpetrators who were ultimately sentenced took several years in each of the cases. The low proportion of charges and the long time interval needed for a judgement in the Hungarian legal system demonstrate the challenges associated with successful prosecution. It is of great importance to organize more family planning centers for young families, children and teenagers to have access to appropriate health care services and suitable education to protect them from sexual abuse.

## 5. Conclusions

The prevention of sexual abuse is the responsibility not only of parents, but law enforcement agencies, health professionals and educators. In Hungary the training of professionals in the recognition and reporting of CSA is an urgent matter, the absence of a legal obligation to report needs reconsideration. The results stress the importance of the need for a public awareness campaign regarding the vulnerability of children of ages to sexual victimization with a focus on the development of primary prevention programs. Professionals in health care, child protection, mental health and law enforcement would all benefit from efforts to raise their awareness of child sexual abuse and what they can do in their professional role to address abuse when suspected.

## Figures and Tables

**Table 1:** Characteristics of the victims (n = 426).

Characteristics	Category	Male N (%)	Female N (%)	Total N (%)
Age (years)	<10	12 (46.2)	56 (14.0)	68 (16.0)
	11–14	10 (38.5)	178 (44.5)	188 (44.1)
	>14	4 (15.4)	166 (41.5)	170 (39.9)
Education	Preschool	4 (15.4)	22 (5.5)	26 (6.1)
	Pupil	19 (73.1)	224 (56.0)	243 (57.0)
	Other	3 (11.5)	154 (38.5)	157 (36.9)
Perpetrator	Father	1 (3.8)	12 (3.0)	13 (3.1)
	Stepfather	0	24 (6.0)	24 (5.6)
	Stepbrother	3 (11.5)	2 (0.5)	5 (1.2)
	Cousin	3 (11.5)	2 (0.5)	5 (1.2)
	Grandfather	1 (3.8)	0	1 (0.2)
	Other relative	0	31 (7.8)	31 (7.3)
	Familiar	10 (38.5)	189 (47.3)	199 (46.7)
	Stranger	8 (30.8)	140 (35.0)	148 (34.7)
Escorter	Alone	0	43 (10.8)	43 (10.1)
	Mother	9 (34.6)	96 (24.0)	105 (24.6)
	Parents	6 (23.1)	21 (5.3)	27 (6.3)
	Other relative	2 (7.7)	9 (2.3)	11 (2.6)
	Familiar	4 (15.4)	18 (4.5)	22 (5.2)
	Paramedical officer	0	13 (3.3)	13 (3.1)
	Police	5 (19.2)	200 (50.0)	205 (48.1)

**Table 2:** Characteristics of CSA cases.

Characteristics	Category	Male N (%)	Female N (%)	Total N (%)	*p* Value
Time of the examination	Immediate	2 (7.7)	98 (24.5)	100 (23.5)	* *p* = 0.002
	Within 72 h	5 (19.2)	150 (37.5)	155 (36.4)	
	More than 72 h	19 (73.1) *	152 (38.0)	171 (40.1)	
Type of abuse	Vaginal penetration	0	219 (54.8)	219 (51.4)	* *p* < 0.001
	Sexual perversion	14 (53.8)	164 (41.0)	178 (41.8)	
	Anal penetration	12 (46.2) *	14 (3.5)	14 (3.3)	
	Physical abuse	0	15 (3.75)	15 (3.5)	
Pregnancy Test	Negative	-	5 (1.3)	-	-
	Positive	-	12 (3.0)	-	
	Not done	-	383 (95.8)	-	
Sperm diagnostic	Positive	1 (3.8)	117 (29.3)	118 (27.7)	* *p* = 0.005
	Negative	25 (96.2) *	283 (70.8)	308 (72.3)	
Occurrence	single	18 (69.2)	320 (80.0)	338 (79.3)	* *p* = 0.189
	multiple	8 (30.8)	80 (20.0)	88 (20.7)	
Location of abuse	Familiar home	2 (7.7)	81 (20.3)	83 (19.5)	
	Car	0	13 (3.3)	13 (3.1)	
	Children’s refuge	4 (15.5)	13 (3.3)	17 (4.0)	
	School	3 (11.5)	21 (5.3)	24 (5.6)	
	Bar, disco	0	34 (8.5)	34 (8.0)	
	Public space	4 (15.4)	57 (14.3)	61 (14.3)	
	Forest, field	3 (11.5)	44 (11.0)	47 (11.0)	
	Victim’s home	9 (34.6)	123 (30.8)	132 (31.0)	
	other	1 (3.8)	14 (3.5)	15 (3.5)	
Seasonal occurrence	Spring	6 (23.1)	112 (28.0)	118 (27.7)	
	Summer	10 (38.5)	117 (29.3)	127 (29.8)	
	Autumn	5 (19.2)	84 (21.0)	89 (20.9)	
	Winter	5 (19.2)	87 (21.8)	92 (21.6)	
Diurnal occurrence	Morning	5 (19.2)	54 (13.5)	59 (13.8)	
	Afternoon	11 (42.3)	132 (33.0)	143 (33.6)	
	Evening	7 (26.9)	150 (37.5)	157 (36.9)	
	Night	3 (11.5)	64 (16.0)	67 (15.7)	

## Geolocation Info

The study took place in four cities (University Teaching Hospital of Nyíregyháza, University Teaching Hospital of Fehérgyarmat, City Hospital of Mátészalka and the City Hospital of Kisvárda) in Szabolcs-Szatmár-Bereg County in the northeastern part of Hungary. The county has approximately 585,000 inhabitants of the 9,900,000 inhabitants of Hungary. Hungary is located in Middle Eastern Europe, between Austria and Romania.
